# Depressive symptoms associated with COVID-19 preventive practice measures, daily activities in home quarantine and suicidal behaviors: Findings from a large-scale online survey in Bangladesh

**DOI:** 10.1186/s12888-021-03246-7

**Published:** 2021-05-26

**Authors:** Md. Saiful Islam, Rafia Tasnim, Md. Safaet Hossain Sujan, Most. Zannatul Ferdous, Md. Tajuddin Sikder, Jakir Hossain Bhuiyan Masud, Sourav Kundu, Promi Tahsin, Abu Syed Md. Mosaddek, Mark D. Griffiths

**Affiliations:** 1 Department of Public Health and Informatics, Jahangirnagar University, Savar, Dhaka, 1342 Bangladesh; 2 Centre for Advanced Research Excellence in Public Health, Savar, Dhaka, 1342 Bangladesh; 3 Quest Bangladesh Biomedical Research Center, Lalmatia, Dhaka, 1207 Bangladesh; 4 Public Health Informatics Foundation (PHIF), Mirpur, Dhaka, 1216 Bangladesh; 5 Advanced Institute of Industrial Technology, Shinagawa City, Tokyo, 140-0011 Japan; 6 Department of Pharmacology, Uttara Adhunik Medical College, Uttara, Dhaka, 1230 Bangladesh; 7 International Gaming Research Unit, Psychology Department, Nottingham Trent University, 50 Shakespeare Street, Nottingham, NG1 4FQ UK

**Keywords:** Depressive symptoms, Suicidal behaviors, Mental health, Home quarantine, COVID-19

## Abstract

**Background:**

The world is facing a public health emergency situation caused by the COVID-19 pandemic. Psychological wellbeing among individuals worldwide has been negatively affected by the pandemic especially in low- and middle-income countries such as Bangladesh. The present study aimed to assess the estimate of depressive symptoms and investigated its associations with COVID-19 preventive practice measures, daily activities in home quarantine, and suicidal behaviors in a large-scale Bangladeshi online survey.

**Methods:**

An online-based cross-sectional survey was widely distributed to Bangladeshi citizens. A total of 13,654 participants (61.0% male; mean age = 24.0 years [SD = 6.0]; age range 18–65 years) completed the survey between May and June (2020). The survey included socio-demographics and COVID-19-related questions, along with lifestyle, suicidal, and psychometric measures. Hierarchical regression was performed to determine significant associations between depression and examined variables.

**Results:**

The estimate of depressive symptoms during the COVID-19 pandemic was 43.5%. Based on hierarchical regression analysis, depression was significantly associated with not engaging in COVID-19 preventive measures, daily activities in home quarantine (e.g., playing videogames), and suicidal behaviors.

**Conclusions:**

Depressive symptoms appeared to be high during the COVID-19 pandemic in Bangladesh. To fight against the pandemic, mental health issues as well as physical health issues need to be taken into consideration.

**Supplementary Information:**

The online version contains supplementary material available at 10.1186/s12888-021-03246-7.

## Background

Currently, the world is facing a public health emergency situation of international concern due to the novel coronavirus-2019 (COVID-19) [1]. COVID-19 originated in Wuhan (China) at the end of 2019 as viral pneumonia. By March 2020, the World Health Organization (WHO) declared the disease to be a pandemic as it quickly spread through the rest of the world. This deadly virus has already infected over 155 million individuals and killed over 3.2 million people globally [2]. In Bangladesh (where the present study was carried out), the virus was first reported on March 8, 2020 [3, 4]. Since then, the virus quickly spread and there have now been more than 767,000 cases and over 11,700 deaths (May 5, 2021) [5].

The Bangladesh government enforced partial (zonal) lockdown, home quarantine, and restricted travel to suppress the spread of the virus [6], and COVID-19 has caused public panic and stress on mental health [7]. Pandemic issues such as spatial distancing, isolation, and quarantine, as well as social and economic consequences, have led to depression, frustration, fear, grief, anger, shame, desperation, boredom, stress, and panic [6, 8–10]. These are common mental health problems that many individuals will experience during and after the crisis [11], and which can also play a dynamic role in the ideation of suicide [12]. During and after an outbreak and as a consequence of isolation and quarantine, suicidal ideation among the population increases [13]. Strict social isolation and mass home quarantine have significant implications for adolescents and adults [14], which could (and has) lead to suicide [12, 15, 16].

Depression is a significant psychiatric illness affecting more than 264 million people around the world [17]. It is a common psychological disorder and comprises depressed mood, loss of interest or pleasure, guilt or low self-worth, troubled sleeping, loss of appetite, low energy, and low concentration [18]. Depressive disorders are common forms of mental disorders, which are frequently reported across the lifespan from adolescence to old age all over the world [19].

Depression has been defined as when an individual has experienced a depressed mood or lost interest or enjoyment in everyday activity for at least two weeks and has a majority of specific symptoms such as negative detriments to sleep, eating, energy, concentration and/or self-worth [20]. Individuals’ psychological wellbeing correlates with daily activities [21]. Such behaviors frequently disturb in sporting and physical activity, as well as increase the risk of depression [22]. Moreover, problematic internet use and gaming addiction may also increase depression during the pandemic [23]. However, the fear of being infected and family infection, and the stress of testing positive for COVID-19 can also increase the level of depression among the general population [24].

To date, no study in Bangladesh has investigated the associations of depressive symptoms with COVID-19 preventive practice measures and daily activities in home quarantine during the pandemic among the general population. Therefore, the present study assessed the estimate of depressive symptoms and investigated its association with COVID-19 preventive practice measures, daily activities in home quarantine, and suicidal behaviors during the COVID-19 pandemic period among the general population of Bangladesh. The findings will be of use to healthcare providers and health policymakers in Bangladesh given that nations will be living with COVID-19 for years to come.

## Methodology

### Study design and participants

The present study was a cross-sectional survey conducted between May and June (2020). The study comprised 13,654 participants who resided in Bangladesh. The study’s target population was Bangladeshi citizens who were house-bound in Bangladesh during the COVID-19 pandemic. The inclusion criteria included being (i) aged 18 years or older, (ii) able to read Bangla, and (iii) able to complete the entire survey. The exclusion criteria included being aged under 18 years and not completing the entire survey.

### Data collection procedure

A semi-structured questionnaire was employed to collect data during the survey. An internet-based survey was conducted using a link via *Google Forms.* A pilot test was performed on 150 individuals to ensure all questions were appropriate and easy to understand. To enable a quick response and to cover all regions in Bangladesh, a voluntary team with more than 200 members were recruited from different parts of the country. This team significantly contributed to collect a large sample by sharing the survey link in their different online platforms (e.g., *Facebook, Messenger, WhatsApp,* etc.). Initially, 14,353 participants submitted the survey form after obtaining informed consent. Of these, 14,095 participants completed the entire survey voluntarily and anonymously. After eliminating incomplete surveys along with participants below 18 years, 13,654 valid surveys were included in final analysis.

### Measures

A self-report survey comprised five sections including socio-demographic information, COVID-19 related questions, as well as lifestyle, suicidal, and psychometric measures. Details of these are provided below.

#### Socio-demographic measures

Socio-demographic information was collected during the survey including age, sex, educational qualifications, marital status, family type, monthly family income, and residence (urban/rural). Socio-economic status (SES) was categorized into three classes: lower, middle, and upper based on monthly family income of less than 15,000 Bangladeshi Taka (BDT) ≈ 177 US$, 15,000–30,000 BDT ≈ 177–354 US$, and more than 30,000 BDT ≈ 354 US$, respectively [25, 26].

#### COVID-19 related measures

The COVID-19 related measures were recorded during survey asking ‘yes/no’ questions including: *Has anyone in your family been infected with COVID-19?*, *Have you had a COVID-19 detection test?*, *Have you had a fear that COVID-19 could infect you?*, *Is the pandemic affecting your family income?*, *Has anyone in your family lost their job during the pandemic?*, and *Has there been a scarcity of food for your family during the pandemic?* Likewise, ‘yes/no’ questions regarding preventive practice towards COVID-19 were asked during the survey based on the WHO guidelines [27] (e.g., using handkerchief/tissue during sneezing or coughing, sniffing the nose by the fold of elbows while sneezing and coughing, discharging cough/sputum in a proper place, washing hands with soaps or other cleaners, using a face mask while outside, discharging tissue in a proper place, maintaining spatial distance, and maintaining hygiene).

#### Lifestyle and activities during home quarantine

Lifestyle measures were collected during the survey by asking questions concerning physical exercising (yes/no), average number of daily sleeping hours, average number of internet browsing hours daily, and cigarette smoking (yes/no) during the COVID-19 pandemic. Moreover, some additional ‘yes/no’ questions were asked regarding which daily activities were engaged in while in home quarantine (e.g., reading academic books or others, taking online classes, video gaming, online communication, watching movies, serials, short films, etc., doing household chores, and spending time talking with family members). Sleeping hours were classified into three categories on the basis of average daily sleeping hours and classed as normal (7-9 h), less than average (< 7 h), or more than average (> 9 h) based on previous literature [28, 29].

#### Suicidal measures

Suicidal measures were recorded during the survey by asking questions concerning past suicidal thoughts, suicide attempt history, and family member suicidal history. With regard to assessing suicidal ideation, participants were asked the question: *“During the COVID-19 pandemic, have you ever seriously thought about killing yourself?”* [30]. The construction of this question was based on previous studies that assessed suicidal ideation [31–34]. Family history of suicide was assessed using the question, “*Has anyone in your family committed suicide?*”. In addition, the question “*Have you ever attempted to kill yourself?*” was asked during the survey to assess suicide attempt history. These questions were used in recent studies [30, 34, 35].

#### Patient health questionnaire (PHQ-9)

The PHQ-9 is the most widely used self-reported screening tool for assessing the severity of depressive symptoms [36]. This scale comprises nine items with a four-point Likert scale ranging from 0 (“*Not at all”*) to 3 (“*Nearly every day”*). Each item refers to problems experienced including issues with sleep, exhaustion, changes in appetite, difficulties with concentration, and suicidal thoughts are assessed over the past two weeks (e.g., *“Little interest or pleasure in doing things”*). The present study used the validated Bangla version of the PHQ-9 questionnaire to investigate the level of participants’ depressive symptoms [37], which has been used in so many recent studies in Bangladesh [38–40]. The total score was calculated by summing the raw scores of each item, with a higher score indicating a greater level of depression. The levels of depressive symptoms were classified into five groups as minimal, mild, moderate, moderately severe, and severe based on scoring 0–4, 5–9, 10–14, 15–19, and 20–27, respectively. In present study, the PHQ-9 scale was found to have very good reliability (Cronbach’s alpha = 0.88).

### Statistical analysis

Some basic statistics (i.e., frequencies, percentages, means, standard deviations, chi-square tests, etc.) were investigated. In addition, *t*-tests or one-way ANOVA tests were performed to determine significant relationships concerning the mean depression scores with all examined variables applying Bonferroni correction (by dividing *p*-value significance threshold into the number of independent variables [0.05/30 = 0.002]). Finally, COVID-19 related variables, lifestyle and activities in home quarantine, and suicidal behaviors that significantly differed in terms of depression scores were included into hierarchical regression analysis with depression as the dependent variable. The analyses were performed using SPSS software version 25.0.

### Ethics

The study was conducted in accordance with the Institutional Research Ethics guidelines and ethical guidelines involving human participation (i.e., Helsinki Declaration). Formal ethical approval was granted by the Ethical Review Committee, Uttara Adhunik Medical College (Ref. No.: UAMC/ERC/ 13/2020). After providing informed consent, participants completed the survey. The consent form specifically outlined the aims, objectives, nature, and procedure of the study, and clearly stated the right to withdraw data from the survey at any time. All participants were assured that all their data would be anonymous and confidential because no identifying information was recorded (e.g., name, mobile number, address, etc.).

## Results

### General characteristics of participants

A total of 13,654 participants were included in the final analysis. Of these, 61.4% were male, the mean age was 24.0 years (SD = 5.9), and age ranged from 18 to 65 years, and almost all participants were unmarried (82.4%). The majority had bachelor degree level of education (67.6%), came from nuclear family (78.2%), were higher SES (44.8%), and were from urban areas (62.0%) (Table 1). Two-thirds of participants (67.8%) slept in a normal range (7–9 h) and most (84.0%) did not smoke cigarettes. Additionally, a substantial majority of participants used internet more than six hours daily (31.4%), and 48.9% did not engage in physical exercise during the quarantine (Table 2).

**Table 1:** General characteristics of participants and their association with depression

Variables	Depression				Total ***N*** = 13,654		Chi-square test ***p***-value
	*Negative*^a^		*Positive*^b^				
	*n*	(%)	*n*	(%)	*n*	(%)	
**Sex**							< 0.001
Male	4963	(59.2)	3419	(40.8)	8382	(61.4)	
Female	2756	(52.3)	2516	(47.7)	5272	(38.6)	
**Age**							0.067
Young (18–25 years)	6068	(56.9)	4588	(43.1)	10,656	(78.0)	
Older (> 25 years)	1651	(55.1)	1347	(44.9)	2998	(22.0)	
**Educational qualification**							< 0.001
Intermediate (11–12) or bellow	1242	(49.2)	1282	(50.8)	2524	(18.5)	
Bachelor	5405	(58.6)	3821	(41.4)	9226	(67.6)	
Higher education (above bachelor)	1072	(56.3)	832	(43.7)	1904	(13.9)	
**Marital status**							< 0.001
Unmarried	6468	(57.5)	4781	(42.5)	11,249	(82.4)	
Married	1216	(52.0)	1122	(48.0)	2338	(17.1)	
Divorced	35	(52.2)	32	(47.8)	67	(.5)	
**Family type**							0.287
Nuclear	6010	(56.3)	4666	(43.7)	10,676	(78.2)	
Join	1709	(57.4)	1269	(42.6)	2978	(21.8)	
**Socio-economic status**							0.310
Lower	1516	(57.9)	1104	(42.1)	2620	(19.2)	
Middle	2759	(56.2)	2153	(43.8)	4912	(36.0)	
Upper	3444	(56.3)	2678	(43.7)	6122	(44.8)	
**Residence**							0.410
Rural area	2912	(56.1)	2280	(43.9)	5192	(38.0)	
Urban area	4807	(56.8)	3655	(43.2)	8462	(62.0)	

*BDT* Bangladeshi Taka

^a^Negative depression using PHQ-9 score (< 10)

^b^Positive depression using PHQ-9 score (≥ 10)

**Table 2:** Descriptive analysis of each variable and association with depression scores

Variables	Total N = 13,654		Depression (Mean = 9.5; SD = 6.7)			
	***n***	(%)	***Mean***	***(SD)***	***t/F***	***p***-value
***COVID-19 related factors***						
**Has anyone in your family been infected with COVID-19?**						
Yes	417	(3.1)	12.2	(5.9)	94.41	< 0.001
No	13,237	(96.9)	9.0	(6.7)		
**Have you had a COVID-19 detection test?**						
Yes	716	(5.2)	11.1	(6.2)	73.58	< 0.001
No	12,938	(94.8)	8.9	(6.7)		
**COVID-19 tests results**						
Positive	286	(5.1)	11.6	(6.1)	26.52	< 0.001
Negative	5297	(94.9)	9.5	(6.7)		
**Have you had a fear that COVID-19 could infect you?**						
Yes	9372	(68.6)	9.6	(6.7)	167.56	< 0.001
No	4282	(31.4)	8.0	(6.7)		
**Is the pandemic affecting your family income?**						
Yes	9394	(68.8)	9.2	(6.8)	17.63	< 0.001
No	4260	(31.2)	8.7	(6.5)		
**Has anyone in your family lost their job during the pandemic?**						
Yes	2547	(18.7)	10.5	(6.7)	149.02	< 0.001
No	11,107	(81.3)	8.7	(6.7)		
**Has there been a scarcity of food for your family during the pandemic?**						
Yes	3337	(24.4)	10.5	(6.8)	201.25	< 0.001
No	10,317	(75.6)	8.6	(6.6)		
**Using handkerchief/tissue during sneezing or coughing**						
Yes	11,504	(84.3)	8.9	(6.7)	37.46	< 0.001
No	2150	(15.7)	9.9	(6.8)		
**Sniffing the nose by the fold of elbows while sneezing and coughing**						
Yes	11,703	(85.7)	9.0	(6.7)	12.95	< 0.001
No	1951	(14.3)	9.6	(6.8)		
**Discharging cough/sputum in a proper place**						
Yes	11,997	(87.9)	8.8	(6.8)	98.50	< 0.001
No	1657	(12.1)	10.6	(6.1)		
**Washing hands with soaps or other cleaners**						
Yes	12,910	(94.6)	8.9	(6.7)	101.96	< 0.001
No	744	(5.4)	11.5	(5.9)		
**Using a face mask while outside**						
Yes	12,986	(95.1)	9.0	(6.7)	23.75	< 0.001
No	668	(4.9)	10.3	(6.6)		
**Discharging tissue in a proper place**						
Yes	12,585	(92.2)	9.0	(6.7)	29.97	< 0.001
No	1069	(7.8)	10.1	(6.4)		
**Maintaining spatial distance**						
Yes	12,654	(92.7)	8.9	(6.7)	56.04	< 0.001
No	1000	(7.3)	10.6	(6.8)		
**Maintaining hygiene**						
Yes	12,265	(89.8)	9.0	(6.7)	10.70	0.001
No	1389	(10.2)	9.6	(6.7)		
***Lifestyle and activities in home quarantine***						
**Sleeping hours**						
< 7 h	2670	(19.6)	10.3	(6.7)	267.22	< 0.001
> 9 h	1722	(12.6)	11.7	(6.9)		
7–9 h	9262	(67.8)	8.2	(6.5)		
**Smoking status**						
Yes	2179	(16.0)	9.8	(6.6)	29.21	< 0.001
No	11,475	(84.0)	8.9	(6.7)		
**Internet using hours**						
< 2 h	1665	(12.2)	7.9	(6.1)	114.01	< 0.001
2–4 h	4245	(31.1)	8.0	(6.4)		
5–6 h	3456	(25.3)	9.2	(6.4)		
> 6 h	4288	(31.4)	10.4	(7.2)		
**Attending online classes**						
Yes	4853	(35.5)	8.9	(6.8)	3.34	0.068
No	8801	(64.5)	9.1	(6.6)		
**Playing videogames**						
Yes	4648	(34.0)	9.5	(6.8)	28.35	< 0.001
No	9006	(66.0)	8.8	(6.7)		
**Engaging in online communication (chat, video calling)**						
Yes	8807	(64.5)	9.2	(6.7)	12.79	< 0.001
No	4847	(35.5)	8.8	(6.7)		
**Watching movies, serials, short films, etc.**						
Yes	10,620	(77.8)	9.1	(6.7)	3.18	0.075
No	3034	(22.2)	8.9	(6.8)		
**Studying**						
Yes	7144	(52.3)	8.2	(6.6)	224.00	< 0.001
No	6510	(47.7)	9.9	(6.7)		
**Engaging in physical exercise**						
Yes	6971	(51.1)	8.4	(6.4)	144.86	< 0.001
No	6683	(48.9)	9.8	(7.0)		
**Doing household chores**						
Yes	10,481	(76.8)	8.8	(6.6)	60.22	< 0.001
No	3173	(23.2)	9.9	(6.9)		
**Spending time talking with family members**						
Yes	12,114	(88.7)	8.8	(6.6)	128.91	< 0.001
No	1540	(11.3)	10.9	(7.1)		
***Suicidal behaviors***						
**Suicide ideation**						
Yes	1097	(8.0)	15.2	(7.1)	1082.00	< 0.001
No	12,557	(92.0)	8.5	(6.4)		
**Past suicidal thoughts**						
Yes	1865	(13.7)	13.4	(7.2)	962.01	< 0.001
No	11,789	(86.3)	8.4	(6.4)		
**Suicidal attempt history**						
Yes	1040	(7.6)	13.5	(7.0)	521.51	< 0.001
No	12,614	(92.4)	8.7	(6.6)		
**Family suicide history**						
Yes	499	(3.7)	11.8	(6.4)	84.58	< 0.001
No	13,155	(96.3)	9.0	(6.7)		

Over two-thirds had a fear that “the coronavirus could infect me” (68.6%). Additionally, two-thirds had a negative impact (decreased comparing pre-COVID-19 situation) on their family income (68.8%), one-fifth of participants had family members who lost their jobs because of the pandemic (18.7%), and a quarter said there was a scarcity of food during the pandemic (24.4%).

### Estimates of depression and suicidal behaviors

Analysis demonstrated a severity depression distribution of 30.5% minimal depression, 26.1% mild depression, 21.4% moderate depression, 14.5% moderately severe depression, and 7.5% severe depression. Based on PHQ-9 cutoff (≥ 10), the estimates of moderate to severe depression were 43.5% with a mean score of 9.5 (SD = 6.7). Almost one in ten participants reported suicidal ideation during the COVID-19 pandemic (8.0%), and 13.7% participants reported past suicidal thoughts prior to the COVID-19 pandemic. Additionally, a minority reported prior suicidal attempts (7.6%), and 3.7% reported a family history of suicide.

### Group differences analysis

With regard to socio-demographic variables, the proportion of moderate to severe depression was significantly higher among (i) females vs. males (47.7% vs. 40.8%, *p* < 0.001), (ii) participants with comparatively lower vs. higher education (50.8% vs. 41.4%, *p* < 0.001), and (iii) married vs. unmarried participants (48.0% vs. 42.5%, *p* < 0 .001) (Table 1).

With regard to COVID-19 related variables, the mean depression score was significantly higher among (i) participants with COVID-19 infected family members, (ii) participants with COVID-19 detection test, (iii) participants with COVID-19 positive result, (iv) participants who had a fear of infection with COVID-19, (v) participants with decreased family income due to pandemic, (vi) participants with family members who lost their jobs during the pandemic, and (vii) participants who had a scarcity of food during the pandemic (Table 2).

The mean depression score was significantly higher among participants who did not engage in good COVID-19 preventive practices vs. those who did, including (i) using handkerchief/tissue during sneezing or coughing, (ii) covering mouth and nose with elbows when coughing or sneezing, (iii) discharging cough/sputum in proper place, (iv) washing hands with soap or other cleaners, (v) using a mask while outside, (vi) discharging tissue in proper place, (vii) maintaining spatial distance, and (vii) maintaining hygiene (Table 2).

With regard to the measures of lifestyle and activities in home quarantine, the mean score was significantly higher among participants who (i) slept more (> 9 h), (ii) were smokers, (iii) used the internet > 6 h daily, (iv) played videogames, (v) engaged in online communication (chat, video calling), (iv) did not read academic books, (vii) did not engage in physical exercise, (viii) did not do household chores, and (ix) did not spend time with their family members (Table 2). With regard to suicidal behavior, the mean depression score was significantly higher among participants with (i) suicidal ideation during the pandemic, (ii) suicidal thoughts prior to the pandemic, (iii) suicidal attempt history and, (iv) a family suicide history (Table 2).

### Hierarchical regression predicting depression

Table 3 summarizes the results of hierarchical regression analysis. Factors that were statistically significant in the group difference analyses (*t*-tests and ANOVA) were included in a hierarchical regression analysis. COVID-19-related variables were included in Block 1, lifestyle and activities during home quarantine comprised Block 2, and suicidal behavior measures comprised Block 3 (see Table 3). Depression was positively associated with having COVID-19 infected family members, having fear of COVID-19 infection, having decreased family income due to the impact of COVID-19, having family members who lost their jobs during the pandemic, and having a scarcity of food during the pandemic. Furthermore, participants who did not maintain COVID-19 prevention measures were significantly more likely to be depressed than those who did maintain them including discharging cough/sputum in a proper place and washing hands with soaps, sanitizer or other cleaners. Depression was also associated with less or more sleep than normal, being heavy internet users, playing videogames, not studying, having suicidal ideation, and having prior suicidal thoughts. The regression model predicted 16% of the variance in depression (*F*_(28,5554)_ = 39.67).

**Table 3:** Hierarchical regression analysis predicting depression

Model	B	SE	β	***t***	ΔR^**2**^
**Block 1 – COVID-19 related variables** (*F*_(15,5567) = _21.67; *p* < 0.001)					0.06
Has anyone in your family been infected with COVID-19?^a^	−1.56	0.42	−0.05	−3.71*	
Have you had a COVID-19 detection test?^a^	−0.44	0.28	−0.02	−1.60	
COVID-19 test results^b^	0.32	0.43	0.01	0.75	
Have you had a fear that COVID-19 could infect you?^a^	−1.33	0.18	−0.10	−7.38*	
Is the pandemic affecting your family income?^a^	0.86	0.19	0.06	4.42*	
Has anyone in your family lost their job during the pandemic?^a^	−1.10	0.22	−0.07	−4.94*	
Has there been a scarcity of food for your family during the pandemic?^a^	−0.88	0.20	−0.06	−4.37*	
Using handkerchief/tissue during sneezing or coughing^a^	−0.33	0.28	−0.02	−1.20	
Sniffing the nose by the fold of elbows while sneezing and coughing^a^	−0.61	0.26	−0.03	−2.33	
Discharging cough/sputum in a proper place^a^	2.10	0.27	0.12	7.78*	
Washing hands with soaps or other cleaners^a^	1.65	0.37	0.07	4.49*	
Using a face mask while outside^a^	−0.81	0.45	−0.03	−1.80	
Discharging tissue in a proper place^a^	−0.25	0.38	−0.01	−0.66	
Maintaining spatial distance^a^	0.72	0.41	0.03	1.74	
Maintaining hygiene^a^	−0.19	0.35	−0.01	− 0.55	
**Block 2 – Lifestyle and activities in home quarantine** (*F*_(24,5558) =_ 27.41; *p* < 0.001)					0.05
Sleeping hours^c^	−0.93	0.10	−0.12	−9.28*	
Smoking status^a^	−0.17	0.22	−0.01	−0.77	
Internet using hours^d^	0.70	0.09	0.11	8.09*	
Playing videogames^a^	−0.80	0.18	−0.06	−4.44*	
Engaging in online communication (chat, video calling)^a^	−0.06	0.19	0.00	−0.30	
Studying^a^	1.05	0.17	0.08	6.14*	
Engaging in physical exercise^a^	0.53	0.18	0.04	2.94	
Doing household chores^a^	−0.41	0.23	−0.03	−1.81	
Spending time talking with family members^a^	0.75	0.29	0.04	2.63	
**Block 3 – Suicidal behaviors** *F*_(28,5554)_ = 39.67, *p* < 0.001					0.06
Suicidal ideation^a^	−3.63	0.31	−0.16	−11.92*	
Past suicidal thoughts^a^	−2.36	0.29	−0.12	−8.09*	
Suicidal attempt history^a^	−1.00	0.34	−0.04	−2.92	
Family suicide history^a^	0.39	0.39	0.01	1.01	

*B* unstandardized regression coefficient, *SE* standard error, *β* standardized regression coefficient; ^a^1 = Yes, 2 = No; ^b^1 = Positive, 2 = Negative; ^c^1 = < 7 h, 2 = > 9 h, 3 = 7–9 h; ^d^1 = < 2 h, 2 = 2–4 h, 3 = 5–6 h, 4 = > 6 h; *R*^2^_Adj_ = 0.16; *p < 0.001

## Discussion

Following the severe acute respiratory syndrome (SARS) outbreak in 2003 [41], the COVID-19 pandemic is the biggest ever viral pandemic and has quickly become the biggest public health issue with significant effect on mental health. The present study found the estimate of moderate to severe depression to be 43.5%, and was significantly associated with being female (47.7%), having lower education [as opposed to higher education] (50.8%), and being married (48.0%). Based on hierarchical regression analysis, depression was associated with disturbed sleep, increased hours using the internet, not engaging in COVID-19 related measures, specific activities during home quarantine (e.g., playing videogames), and suicidal behaviors.

### Comparison with other studies

Despite wide variation among community settings, cultural backgrounds, participant perspectives, and survey methodologies, the results reported here broadly concur with previous works, including previous surveys, review papers, and meta-analyses that examined (i) depression, (ii) depression and lifestyle/behaviors, and (iii) suicidal behaviors for general population.

According to the findings of the present study, females were more likely to be depressed than males, and being female has been found as a risk factor for depression in almost all previous studies [6, 42–44]. However, a study in Pakistan found no significant relationship between sex and depression [45]. The findings relating to levels of moderate to severe depression were significantly higher among those who were married (48.0%) or divorced (47.8%) compared to unmarried participants (42.5%). The condition of being alone after marital separation often affects mental wellbeing and can result in depression [46, 47], and the present study supports and aligns with such findings. Previous literature has also reported that being married (compared to single, widowed or separated) can be associated with depression [48].

Family members of the participants affected by COVID-19 were more highly depressed than non-affected families. Although this is not surprising, no previous study has reported such an association. Obviously seeing loved ones being infected with COVID-19 may act as a traumatic event for an individual which can be a contributory cause of depression [49]. The level of depression was higher among people who had a fear of being infected by COVID-19 which is line in with previous studies [8]. In extreme cases, the fear of COVID-19 has resulted in suicides in Bangladesh and India highlighting the devastating effect on an individual’s mental well-being [12, 50]. This study found depression was positively associated with decreased family income, job loss, and scarcity of food due to the impact of the COVID-19, and several studies also support this finding [51, 52]. Due to countrywide lockdown and shutdown of non-essential industries, millions of people have lost their jobs. This has led to insecurities about livelihood, poverty, starvation, all of which can be contributing to experiencing depression. As noted earlier, several cases of committing suicide had already occurred in Bangladesh and other Asian countries and such extreme actions clearly demonstrate the impact of job losses on the individual’s psychology [53].

This study established an association between depression and COVID-19 preventive practice measures and found higher depression rates among individuals not engaging in COVID-19 preventive practices including hand hygiene practices, face mask use, use of tissue and adequate disposal of it, and other hygiene practices endorsed by WHO to minimize the exposure from highly infectious virus [54].

Lifestyle and activities in home quarantine were significantly associated with depression. Participants who under slept were less or over slept during the COVID-19 pandemic had higher rates of depression in the present study which is consistent with some preceding research that found a correlation between less sleep and depression [53], and an Asian study showing positive association between depression and sleep disturbance [55]. In the present study, excessive use of the internet and playing videogames were associated with depression a finding that has been reported in many reviews and meta-analyses of internet addiction in the area [22, 56], as well as previous studies in Bangladesh [6, 29, 40]. However, during the current pandemic, participants using the internet heavily were also more likely to read various scary, untrue, and/or deceptive news related to COVID-19 that may also contribute to depression [57]. More specifically, depression was higher among participants not engaged or reading academic books, in contrast with other research showing academic pressure correlates with depression [58, 59].

The estimate of suicidal ideation during the pandemic in the present study was 8.2%, which is slightly higher than the 5% prevalence of suicidal ideation found in a pre-COVID-19 study conducted in Bangladesh [33], and 4.3% in Turkey prior to the COVID-19 pandemic [60]. A more wide ranging survey across 17 countries reported 9.2% prevalence of suicidal ideation [61]. Although none of these latter studies were carried out during the COVID-19 pandemic, the finding of the present study demonstrates that the pandemic may possibly be affecting suicidal thinking, as reported elsewhere [62]. A recent study conducted in Bangladesh reported 12.8% suicidal ideation among university students during the COVID-19 pandemic using the comparable methodology [30]. The percentage of individuals with suicidal ideation and past suicidal thoughts were much higher among depressed individuals than non-depressed individuals during the pandemic, and home quarantine and feeling socially isolated may be a contributory issue among people and a possible vulnerability factor for suicide [14].

To the best of the present authors’ knowledge, the study here is a large-scale study in Bangladesh examining depression in relation to different aspects of pandemic issues among the general population including COVID-19 preventive practice measures, and daily activities in home quarantine during the COVID-19 pandemic. Depressive symptoms were associated with COVID-19 preventive practice measures, daily activities in home quarantine, and suicidal behaviors.

### Limitations

This study has some limitations that must be considered when interpreting the results. Firstly, the study was cross-sectional in nature and was unable to determine causality between any of the variables examined. In this respect, a longitudinal study would be more helpful. Secondly, this research adopted an online self-report methodology that may be open to social desirability and memory recall biases. Being carried out online may have increased the veracity of responses compared to offline and/or face-to-face. Although the study was largescale using a convenience sampling technique, it cannot be considered as nationally representative given the higher proportion of males, bachelor degree level of education, and urban residents that participated, and the low mean age of the sample.

## Conclusions

The COVID-19 pandemic has brought about significant health and life risks in Bangladesh, as well as exacerbating and possibly creating psychological problems such as depression and suicidal ideation among the general population. Some baseline information concerning depression was outlined in this research among general people in Bangladesh. The findings of the present study identified problems related to mental wellbeing and associated risk factors that may be triggering or exacerbating psychological problems as well as identifying individuals who may be at high risk of depression. Such knowledge can be used to inform depression prevention strategies. The effects of pandemics are not equal to all populations, and inequalities always drive psychological problems among individuals. The findings suggest that immediate community-based psychosocial support must be initiated for the general population in Bangladesh considering the nature of the conditions of their mental health in Bangladesh. The findings may contribute as the baseline information for the longitudinal or interventional studies in Bangladesh as well as other lower/ middle income countries.

## Supplementary Information

**Additional file 1.** Details information of volunteers who contributed during the data collection periods.

## Data Availability

The datasets generated and/or analyzed during the current study are not publicly available because the participants didn’t permit to share their data individually rather than an aggregate report but are available from the corresponding author on reasonable request.

## Abbreviations

COVID-19: Coronavirus disease-2019
WHO: World Health Organization
HCWs: Healthcare workers
SES: Socioeconomic status
BDT: Bangladeshi Taka
PHQ: Patient Health Questionnaire
ANOVA: Analysis of variance
SPSS: Statistical Package for the Social Sciences
