# Supplementary material for: Depressive symptoms associated with COVID-19 preventive practice measures, daily activities in home quarantine and suicidal behaviors: Findings from a large-scale online survey in Bangladesh
Source: BMC Psychiatry. 2021 May 26;21:273. doi: 10.1186/s12888-021-03246-7 (PMC8150150; doi:10.1186/s12888-021-03246-7)
Supplement: Supplementary file 1 — Additional file 1. Details information of volunteers who contributed during the data collection periods. [file 12888_2021_3246_MOESM1_ESM.docx]

**Title Page**

**Title: Depressive symptoms associated with COVID-19 preventive practice measures, daily activities in home quarantine and suicidal behaviors: Findings from a large-scale online survey in Bangladesh**

**Author(s):** Md. Saiful Islam^1,2,^*, Rafia Tasnim^1,2^, Md. Safaet Hossain Sujan^1,2^, Most. Zannatul Ferdous^1,3^, Md. Tajuddin Sikder^1^, Jakir Hossain Bhuiyan Masud^3,4^, Sourav Kundu^3,5^, Promi Tahsin^3^, Abu Syed Md. Mosaddek^3,6^, Mark D. Griffiths^7^

**Affiliation(s):**

^1^ Department of Public Health and Informatics, Jahangirnagar University, Savar, Dhaka-1342, Bangladesh

^2^ Centre for Advanced Research Excellence in Public Health, Dhaka, Bangladesh

^3^ Quest Bangladesh Biomedical Research Center, Lalmatia, Dhaka-1207, Bangladesh

^4^ Public Health Informatics Foundation (PHIF), Mirpur, Dhaka-1216, Bangladesh

^5^ Advanced Institute of Industrial Technology, Shinagawa City, Tokyo 140-0011, Japan

^6^ Department of Pharmacology, Uttara Adhunik Medical College, Uttara, Dhaka-1230, Dhaka, Bangladesh

^7^ International Gaming Research Unit, Psychology Department, Nottingham Trent University, 50 Shakespeare Street, Nottingham NG1 4FQ, UK

***Corresponding author**

**Md. Saiful Islam**

Department of Public Health and Informatics, Jahangirnagar University, Savar, Dhaka-1342, Bangladesh, E-mail: [islam.msaiful@outlook.com](mailto:islam.msaiful@outlook.com) or [saiful@phiju.edu.bd](mailto:saiful@phiju.edu.bd), Mobile: +8801779439529, ORCID iD: <https://orcid.org/0000-0003-3979-2423>

Details information of volunteers who contributed during the data collection periods

| Name | | Institution | | Email |
| --- | --- | --- | --- | --- |
| Sk Kamruzzaman | | Patuakhali Science & Technology University | | sk.kamruzzaman1301@gmail.com |
| Alomgir Hossain | | Rajshahi University | | alamgir199817@gmail.com |
| Khairrun Nahar Pinky | | University of Rajshahi | | knpink99@gmail.com |
| Md Nazmus Sakib | | Hamdard University Bangladesh | | nazmusshakib0009@gmail.com |
| Tanziha Yeasmin Toma | | Jahangirnagar University | | Tanzihatoma1613@gmail.com |
| Abdul Ahad Nahin | | Jahangirnagar University | | ahadnahin2015@gmail.com |
| Md. Riad Islam | | Rajshahi University | | riadislam535@gmail.com |
| Md Abid Bin Siddique | | Jahangirnagar University | | abidbinsiddiq6@gmail.com |
| Ikram Hossen | | Jahangir Nagar University | | ikramhossenpranto@gmail.com |
| Fahim Shariar Anter | | Noakhali Science & Technology University | | fahimsontor99@gmail.com |
| Tanmoy Dutta | | Jahangirnagar University | | duttatanmoyju@gmail.com |
| Saleha Begum | | Noakhali Science and Technology University | | mashfikaaymon100@gmail.com |
| Farhan Sarwar | | Jahangirnagar University | | farhansarwar821@gmail.com |
| Rima Basak | | Independent University | | rimabasak381@gmail.com |
| Md Ebrahim Sheikh | | Jagannath University | | ebrahimmkt76@gmail.com |
| Tania Akter Neela | | Noakhali science and technology university | | tanianeela@gmail.com |
| Sanjida Ahmed | | Jahangirnagar University | | sanjidanisa802@gmail.com |
| Tazrin Ahmed Khan | | University of Rajshahi | | tazrinkhan97@gmail.com |
| Sristi Chowdhury | | Noakhali Science and Technology University | | sristichowdhury10@gmail.com |
| Tanjina Yousuf | | Noakhali Science and Technology University | | tanjinayousufjemi@gmail.com |
| Nazmus Sakib | | Jahangirnagar University, Savar, Dhaka | | nazmussakibju2011@gmail.com |
| Shaowni Das | | Jahangirnagar University | | Shaownidasneli@gmail.com |
| Emtius Hasnat Nishat | | Shahidul Chowdhury Engineering College | | emtiushasnat@gmail.com |
| Srabanti Anwar | | Rajshahi University | | shrabananwar@gmail.com |
| Reshma Afroz Rimi | | Jahangirnagar University | | reshmaafroz05@gmail.com |
| Fahmida Alam Chowdhury | | Noakhali Science & Technology University | | fahmidabristyswnstu@gmail.com |
| Ayesha Akter | | University of Rajshahi | | Ayesharahmanasha13@gmail.com |
| Sayma Islam Alin | | Jahangirnagar University | | sayma.alin@phiju.edu.bd |
| Shakib Al Hasan | | Gono Bishwabidyalay | | shakib.2858@gmail.com |
| Saminur Haque | | University of Dhaka | | saminurhaque332@gmail.com |
| Tanjina Akter Shamme | | Jagannath university | | angelshammi78@gmail.com |
| Imran Hosen | | University of Rajshahi | | imranruir14@gmail.com |
| Sajana Tahmid | | North South University | | sejanaq343@gmail.com |
| Samira Akter Siyam | | Govt. College Of Home Economics | | samiraaktersiyam655@gmail.com |
| Tanvir Sarker Tutul | | Jahangirnagar University | | tanvirsarkerju1998@gmail.com |
| Md. Saddam | | Bangabandhu Sheikh Mujibur Rahman Science & Technology University | | saddubmb@gmail.com |
| Sushmita Karmokar | | Noakhali Science and Technology University | | sushmitakarmokar907@gmail.com |
| Md Monirul Islam | | Pharmasia Ltd | | mislamgb@yahoo.com |
| Tasfia Bhuiyan | | Noakhali Science and Technology University | | tasfiaahmedt@gmail.com |
| Rifat Nowshin | | Jahangirnagar University | | rifatnowshin1612@gmail.com |
| Pinon Nath | | Noakhali Science & Technology University | | nathpinon96@gmail.com |
| Md Habibur Rahaman | | Jagannath University | | Habib.jnu1995@gmail.com |
| Mst. Jemi Hkatun | | Jahangirnagar university | | jemikhetlal98@gmail.com |
| Asmaul Husna Ritu | | Jahangirnagar University | | 268husna@gmail.com |
| Jahid Bin Sultan | | Noakhali science and technology university | | zahidsultan470@gmail.com |
| Sabiha Tasnim Reevny | | Jahangirnagar University | | STReevnyJU@gmail.com |
| Mridul Pathan | | Independent University Bangladesh | | mridulpathan17@gmail.com |
| Rezowan Ahmmed | | Khulna University | | 191448@ku.ac.bd |
| Rupa Akter | | Independent University | | [akterrupa919@gmail.com](mailto:akterrupa919@gmail.com) |
| Naimur Rahman | | Jahangirnagar University | | naimur634@gmail.com |
| Md Mujahidul Islam | | Chittagong University | | [mujahid19604032@gmail.com](mailto:mujahid19604032@gmail.com) |
| Most.Israt Jahan | | Noakhali Science and Technology University | | isratmunni2828@gmail.com |
| Afia Ayub | | Tejgaon College, Dhaka | | afia199720122016@gmail.com |
| Rubiya Afrin | | Jahangirnagar University | | afrinrubaiya9@gmail.com |
| A.K.M. Afzal Hossain | | Bangladesh Agricultural University | | a.k.m.afzalhossain92@gmail.com |
| Minhazul Islam Chowdhury | | Southeast University | | sworan007@gmail.com |
| Debashish Paul Deb | | Independent university | | debashispauldeb@gmail.com |
| Md Delwar Hossen | | Independent University, Bangladesh | | delwarhossen1097@yahoo.com |
| Sarup Das | | Uttar Kattali Al-Haj Mostafa Hakim College | | sarupdasnsn@gmail.com |
| Zakia Khanom Tisha | | Jahangirnagar University | | azakiatisha57@gmail.com |
| Mt. Shirajum Monira | | Jagannath University | | shirajumminora.jnu@gmail.com |
| Muhammad Rubel | | Comilla University | | ahmedrubel324@gmail.com |
| Shahadat Hossain Shakil | | Noakhali Scienceand Technology | | shshakil29@gmail.com |
| Shabrina Islam Mim | | University of Barishal | | sabrinamim.law5@gmail.com |
| Kifyat Tasnim | | Jashore University of Science and Technology | | kifyat110196@gmail.com |
| Fahmida Faiza | | International Islamic University Chittagong | | fahmidafaiza921@gmail.com |
| Md Naimul Islam Arif | | University of Rajshahi | | mr.arif3@yahoo.com |
| Sazzad Hossain | | Noakhali Science and Technology University | | sazzadhossainusama@gmail.com |
| Sajjad Bin Sogir | | Jahangirnagar university | | 321mdsajjad@gmail.com |
| Md.Shamsul Haque | | Jagannath University | | shamsulshanto69@gmail.com |
| Md Jabed Hossain | | NSTU | | mdjabedadnan@gmail.com |
| Md Belal Hossen | | Rajshahi University | | belal19599@gmail.com |
| Supti Podder | | University of Dhaka | | suptipodder1998@gmail.com |
| Md.Rezaul Karim | | Noakhali Science &Technology University | | mdrezaul1525@gmail.com |
| Arpita Chakrabarty | | Jahangirnagar University | | a.chakrabarty6116@gmail.com |
| Mahiare-Uz Zaman | | Jahangirnagar University | | mahiershadhin00@gmail.com |
| Tania Akter | | Rajshahi University | | 01745894572@gmail.com |
| Tasmim Hoq | | Independent University Bangladesh | | tasmimhoque789@gmail.com |
| Nusrat Kamal | |  | |  |
| Md Nazmul Hassan | | Comilla University | | nazmulhassan019@gmail.com |
| Md Khaleduzzaman | | Patuakhali Science and Technology University | | iam.khaleduzzaman@gmail.com |
| Md Riazul Islam Sarker | | Jagannath university | | remonahmed78@gmail.com |
| Saifur Rahaman | | Noakhali Science and Technology University | | saifur.nstupharma19@gmail.com |
| Marguba Kamrun | | Jahangirnagor University, Savar , Dhaka | | margubakamrun28@gmail.com |
| Fariba Chowdhury | | Jahangirnagar University | | faribachowdhury12@gmail.com |
| Nishat Anjum Eti | | Independent university | | armstronganjum@gmail.com |
| Nujhat Moonawara | | Dhaka University | | nujhatmuna@gmail.com |
| Amit Kumar Roy | | Beximco Pharmaceuticals Limited | | amitkroy1990@gmail.com |
| Sumaiya Farzana Quaderi | | Global school and College | | sumiju33@gmail.com |
| Sadman Sarar | | Patuakhali Science and Technology University | | sadmansarar777@gmail.com |
| Md. Naeem Islam | | Jahangirnagar University | | mnislam344@gmail.com |
| Maria Meha Promi | | Rajshahi University | | meha.mitsa@gmail.com |
| Md. Saroar Hossen | | Sher-e-Bangla Agricultural University | | sarwar.bd1995@gmail.com |
| Name | **Institution** | | **Email** | |
| Afsara Jahin Rafi | | Jahangirnagar University | | rafijahin@ieee.org |
| Arafat Rahman | | Jahangirnagar University | | a4242r@gmail.com |
| Nadia Akter | | Noakhali Science and Technology University | | Nadia.tazin12@gmail.com |
| Israt Jahan Pinky | | University of Development Alternative | | Israt77uoda@gmail.com |
| Md. Hamidur Rahman | | Asian Disaster Preparedness Center (ADPC) | | sumon.adpc@gmail.com |
| Asma Akter | | National University | | [Asmabinteasmu2@gmail.com](mailto:Asmabinteasmu2@gmail.com) |
| Md Nahid Hassan | | Jahangirnagar University | | mdnahiid6@gmail.com |
| Faria Naznin | | Noakhali Science and Technology University | | farianaznin586@gmail.com |
| Md. Asiqur Rahman | | Sonargaon University | | asiqlaw39du@gmail.com |
| Supriya Saha | | Jashore University of Science & Technology | | Supriyasahajust06@gmail.com |
| Kheirun Nahar Jeni | | Noakhali science and technology University | | aurorajen98@gmail.com |
| Jahanur Biswas | | Jahangirnagar University | | jahanur.biswas23@gmail.com |
| Nazibul Islam | | Shahjalal University of Science and Technology | | shefaul359.islam@gmail.com |
| Md. Ashraful Alam | | Noakhali Science and Technology University | | alamashraf872@gmail.com |
| Tanzila Azad Mow | | Jahangirnagar University | | azadmow03@gmail.com |
| Bijoy Krishna Roy Shuvo | | Jagannath University, Dhaka | | royshuvo168@gmail.com |
| Tanwy Mazumder | | Jagannath University | | tanwymazumder@gmail.com |
| Mahmuda Sultana Mim | | Jahangirnagar University | | mahmudamim772@gmail.com |
| Ridoy Ahamed | | Drug International Limited | | ridoy.ahamed@northsouth.edu |
| Sujeed Debnath | | Jahangirnagar University | | sujitdn48@gmail.com |
| Md. Kamrul Hasan Kayesh | | Bangladesh Agricultural University | | akayes9282@gmail.com |
| Syed Musfiqur Rahman | | Military Institute of Science & Technology | | syedmusfiqur.rahman.17@gmail.com |
| Md Enayatur Rahman | | Jahangirnagar University | | enayatur.ju@gmail.com |
| Khandaker Bushra Rahman | | Noakhali Science and Technology University | | tashmi0101@gmail.com |
| Md. Asif Iqbal | | Bangladesh University of Professionals | | asif92980@gmail.com |
| Md.Naimur Rahman | | North South University | | emonnsu142@gmail.com |
| Sumon Mitra | | IRD Global | | sumonmitra1995@gmail.com |
| Julia Akter Meshu | | University of Rajshahi | | juliaakter.bd@gmail.com |
| Md. Rubel Gazi | | Patuakhali science and technology University | | gmrubel1995@gmail.com |
| Dr. Md. Moksed Ali | | Hamdard University Bangladesh | | drmoksedali@gmail.com |
| Md Zahidur Rahman Arnob | | Jahangirnagar University | | zrarnob@gmail.com |
| Md. Baejid Islam | | Jahangirnagar University | | nafibaejid@gmail.com |
| Md. Ibrahim Khalil | | Noakhali Science and Technology University | | imran.nstu007@gmail.com |
| Akibul Islam Chowdhury | | Noakhali Science and Technology University | | akibul433@gmail.com |
| Sayeda Jahan | | Jahangirnagar University | | sayeda.jahan09@gmail.com |
| Md Mostafa Kamal | | Northeastern Polytechnical University, China | | shuvrobbaju@gmail.com |
| Rejina Akter | | Jahangirnagar University | | rejinadphi@gmail.com |
| Nyeem Ahamed Khan | | Chandlee group of industries | | nyem22khan@gmail.com |
| Md Mustafizur Rahman | | Jahangirnagar University | | fizz1336@gmail.com |
| Md Mehedi Hasan | | Noakhali science and technology univarsity | | rafsanmehedi1001@gmail.com |
| Yeasin Arafath Apu | | Noakhali Science And Technology University | | yeasinsadad52@gmail.com |
| Md. Suliman | | Jagannath University | | sulimansiam82@gmail.com |
| Md Symum Korim | | Rajshahi University | | symumkorim@gmail.com |
| Md.Khaled | | Bangladesh University of Business and Technology | | imkhaled404@gmail.com |
| Sumaiya Akter | | Jahangirnagar University | | sumaiyaabeer1319@gmail.com |
| Ankon Das | | Noakhali Science and Technology University | | ankon2000das@gmail.com |
| Md. Akram Hossain Pavel | | Delta Pharma Limited | | akrampavel50@gmail.com |
| Kobirul Islam | | Dhaka college | | 1996kobirulislam@gmail.com |
| Md. Yeasin Arafat | | Gov't Unani & Ayurvedic Medical College | | arafatimran56@gmail.com |
| Mosa. Nabida Tabassum | | RajshahiUniversity | | nabidanabu25@gmail.com |
